# Successful pulmonary embolectomy for massive pulmonary embolism during pregnancy: a case report

**DOI:** 10.1186/s40981-017-0116-3

**Published:** 2017-08-25

**Authors:** Hiroki Taenaka, Chiyo Ootaki, Chie Matsuda, Yuji Fujino

**Affiliations:** Department of Anesthesiology and Intensive Care, Osaka University Graduate School of Medicine, 2-2 Yamadaoka, Suita, Osaka, 5650871 Japan

**Keywords:** Embolectomy, Fetal monitoring, Intracardiac echography, Labor epidural, Massive pulmonary embolism, Preterm cardiac surgery

## Abstract

**Background:**

Pulmonary embolism (PE) resulting from venous thromboembolism is a leading cause of maternal mortality in pregnancy. In patients with massive PE and hemodynamic instability, the treatment options often considered are thrombolytics, inferior vena caval filters, or embolectomy. We report here the case of a patient with massive PE at 28 weeks’ gestation, who underwent emergency pulmonary embolectomy via cardiopulmonary bypass.

**Case presentation:**

A 35-year old primigravida with a history of massive PE at 25 weeks of gestation was referred to our hospital at 28 weeks of gestation, following treatment failure after insertion of an inferior vena cava filter and heparin administration. Emergency thrombectomy was performed, and intracardiac echography was used for intraoperative fetal heart rate monitoring. However, the patient developed hemodynamic collapse following anesthesia induction; hence, emergency cardiopulmonary bypass (CPB) was performed via median sternotomy. Thrombectomy and tricuspid valve plication were performed under cardiac arrest. After confirming postoperative hemostasis, heparin administration was resumed. At 40 weeks of gestation, labor was induced under epidural analgesia. Both mother and child were discharged with no complications.

**Conclusion:**

In conclusion, intracardiac echography is useful for fetal heart rate monitoring during emergency cardiac surgery in pregnancy. Careful CPB management is important to maintain uteroplacental blood flow. Although there is no consensus on the delivery methods in such cases, epidural analgesia during labor was useful in reducing cardiac load and wound traction.

## Background

Hypercoagulability in pregnancy is a physiological priming for delivery and is associated with an increased risk of venous thromboembolism [1]. Morbidity from venous thromboembolism in pregnant women is five times higher than that in non-pregnant women of similar age [2]. Pulmonary embolism (PE) due to venous thromboembolism is a leading cause of maternal mortality. It was previously thought that the risk of PE is highest during the third trimester and immediately postpartum; however, recent studies have indicated that venous thromboembolism may occur with almost equal frequency in all trimesters. Therefore, PE is a critical complication throughout pregnancy [1, 2]. Management of PE in pregnant women is challenging, due to lack of validated approaches [1], with no established method. Although heparin is the mainstay of therapy for acute venous thromboembolism during pregnancy, thrombolytics, inferior vena caval (IVC) filters, or embolectomy are considered in patients with massive PE and hemodynamic instability [2, 3]. We report the case of a pregnant woman with massive PE that occurred in the second trimester.

## Case presentation

**Fig. 1:**
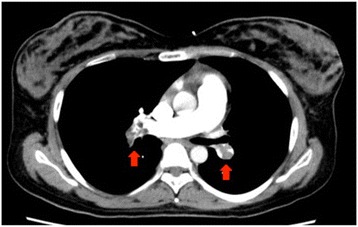
Contrast-enhanced computed tomography of the chest on admission. *Red arrows* show thrombi occupying the pulmonary arteries bilaterally

**Fig. 2:**
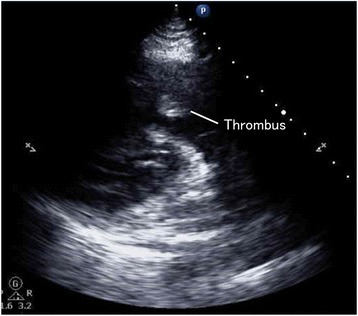
Transthoracic echocardiography. A mobile thrombus is observed in the right ventricle

**Fig. 3:**
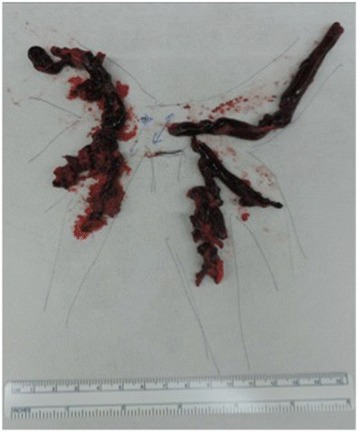
Image showing massive thrombi removed from the pulmonary arteries bilaterally

A 35-year old primigravida (height 150 cm; weight 60 kg) presented with exertional dyspnea at 23 weeks of gestation. A contrast-enhanced computed tomography (CT) scan showed bilateral pulmonary artery thrombi and deep venous thrombus in the lower limb (Fig. 1). A transthoracic echocardiography revealed a right ventricular thrombus (Fig. 2). She was referred to our hospital at 28 weeks of gestation after insertion of IVC filter and heparin administration. Fetal development was normal. Due to the mobility of right ventricular thrombus and the risk of cardiac arrest, an emergency thrombectomy was planned. An intracardiac echography probe (AcuNav™; Siemens AG, Munich, Germany) was inserted via the right femoral vein to monitor the umbilical artery pulse wave via Doppler, in addition to transabdominal fetal monitoring. After induction of anesthesia, the patient’s blood pressure was undetectable; hence, emergency cardiopulmonary bypass (CPB) was performed via median sternotomy, followed by cardiac resuscitation. While switching to extracorporeal circulation approximately 10 min after the hemodynamic collapse, the fetal heart rate decreased to approximately 80 beats per minute. Immediately after establishing extracorporeal circulation, the fetal heart rate recovered rapidly and remained stable throughout the procedure, while the CPB average perfusion pressure was maintained above 70 mmHg. Thrombectomy and tricuspid valve plication were performed under cardiac arrest (Fig. 3). Catecholamines and nitric oxide were administered at a concentration of 20 ppm to treat hypotension and pulmonary hypertension, respectively, during withdrawal from CPB. The patient was admitted to the intensive care unit without chest closure. Total time for surgery, anesthesia, cardiopulmonary, and aortic cross clamp was 345, 483, 208, and 147 min, respectively. The total volume of hemorrhage, transfusion, infusion, urine, and water balance was 1470, 3600, 2150, 1520, and +657 ml, respectively. Chest closure was performed on postoperative day 3 and extubation on postoperative day 6. Heparin administration was resumed after confirming postoperative hemostasis. At 32 weeks of gestation, magnetic resonance imaging (MRI) was performed to assess the fetal brain function; no remarkable changes were observed. The patient was discharged with subcutaneous injection of heparin calcium, due to persistent lower limb thrombus.

She was readmitted at 38 weeks of gestation. Labor was induced at 40 weeks using oxytocin, after discontinuation of heparin. Epidural anesthesia was administered to protect the thoracic wound site and reduce the cardiac load. An epidural catheter was placed between the L3-L4 vertebrae, after confirming the recovery of hemostatic function by measuring activated partial thromboplastin time. Continuous epidural ropivacaine 0.1% with 2 mcg/ml fentanyl was administered through patient controlled epidural analgesia. Analgesia was adequate throughout labor, with no hemodynamic changes. A healthy male infant weighing 3446 g (+1.07 SD) was delivered by vacuum extraction with an Apgar score of 8/8. Total delivery time was 3 h and 37 min, with total blood loss of 928 ml. No remarkable changes were observed in the neonate’s brain on CT and MRI just after delivery. The patient was prescribed oral warfarin for anticoagulation, and both mother and child were discharged without complications.

## Discussion

Heparin is the mainstay of therapy for acute venous thromboembolism during pregnancy; its large molecular weight prevents it from crossing the placenta, reducing the risks of fetal hemorrhage and teratogenesis. However, in nonpregnant patients with massive PE causing hemodynamic instability, thrombolytics, IVC filters, or embolectomy are considered. These strategies may be useful during pregnancy as well [2, 3]. In our patient who developed bilateral massive PE during her second trimester, emergency thrombectomy was performed, as heparin therapy was ineffective.

Cardiac surgery during pregnancy carries significant maternal and fetal risks. Since emergent surgery carries a higher risk of maternal mortality, it is usually considered after failure of medical therapy or in critical cases [2, 4]. Conversely, cardiac surgery can be performed with relative safety owing to recent advances in surgical techniques and CPB [3, 5]. Thromboembolism is fatal in nearly 15% of patients with PE during pregnancy, with two-thirds of deaths occurring within 30 min after the embolic event [6]. In our case, cardiopulmonary arrest following anesthesia occurred likely due to the exacerbation of PE. Therefore, close attention should be paid to sudden hemodynamic changes in cases of PE.

Although mortality is equal among pregnant and non-pregnant women, fetal mortality increases in cases of CPB [7, 8]. CPB may have deleterious effects on the uteroplacental blood flow and the fetus due to activation of inflammatory processes, non-pulsatile flow, hypotension, and hypothermia. The fetal heart rate may decrease during CPB; therefore, uterine tone and fetal heart rate should be closely monitored, especially if the fetus is viable. It is recommended to keep the pump flow rate and perfusion pressure to maintain appropriate uteroplacental blood flow during CPB [9].

In our case, an intracardiac echography probe was inserted via the femoral vein and fixed at the position where the umbilical arterial pulse wave could be monitored by Doppler ultrasound. Although the use of intracardiac echography for fetal heart rate monitoring has not been previously reported, we used it because monitoring fetal heart rate with sterile precautions was necessary during CPB. We carefully inserted it after ensuring the absence of thrombosis and obtained informed consent for this procedure after surgery. It allowed us to monitor the fetal heart rate maintaining the sterile surgical field, which is difficult in conventional abdominal monitoring. The intracardiac echography revealed a decline in the fetal heart rate during maternal cardiopulmonary arrest occurred. Although the obstetrician was on standby in the operating room, emergency delivery was not required as the fetal heart rate recovered following the establishment of CPB and was maintained thereafter. We also successfully maintained the average pump perfusion pressure above 70 mmHg.

Maternal and fetal survival rates after emergency cardiac surgery during pregnancy have improved [5], and the number of patients continuing to parturition after surgery has increased. However, there are few reports on delivery of pregnant women after thoracotomy. In general, exercise and traction on the sternum should be avoided within 3 months of thoracotomy [10]. The vaginal route is therefore generally preferred for delivery of a pregnant woman with cardiac disease. Epidural analgesia inhibits pain during delivery and reduces endogenous catecholamines and peripheral vascular resistance, leading to a decrease in cardiac load [11, 12]. In our case, we successfully placed an epidural catheter to administer analgesia during labor, and vacuum extraction was performed to reduce the load secondary to straining.

## Conclusions

In conclusion, intracardiac echography is useful for fetal heart rate monitoring during emergency cardiac surgery in pregnancy. Careful CPB management is also important to maintain uteroplacental blood flow. Although there is no recommended delivery method for pregnant women after thoracotomy, epidural analgesia during labor was useful in reducing the heart load and wound traction.

## Abbreviations

CPB: Cardiopulmonary bypass
CT: Computed tomography
IVC: Inferior vena caval
MRI: Magnetic resonance imaging
PE: Pulmonary embolism
